# Does Low‐Value Care Explain Health Care Utilization Inequities Among Asian and Latino Populations?

**DOI:** 10.1111/1475-6773.14610

**Published:** 2025-03-20

**Authors:** Sungchul Park, Jie Chen, Arturo Vargas Bustamante, Alexander N. Ortega

**Affiliations:** ^1^ Department of Health Policy and Management, College of Health Science Korea University Seoul Republic of Korea; ^2^ BK21 FOUR R&E Center for Learning Health Systems Korea University Seoul Republic of Korea; ^3^ Department of Health Policy and Management, School of Public Health University of Maryland College Park Maryland USA; ^4^ Department of Health Policy and Management, Fielding School of Public Health, UCLA UCLA Latino Policy and Politics Institute Los Angeles California USA; ^5^ Thompson School of Social Work and Public Health University of Hawai'i at Mānoa Honolulu Hawaii USA

**Keywords:** ethnicity, health disparity, health inequity, low‐value care, race

## Abstract

**Objectives:**

To examine differences in the utilization of low‐value care among Asian and Latino subpopulations compared to the White population.

**Study Setting and Design:**

We analyzed data from a repeated cross‐sectional national survey.

**Data Sources and Analytical Sample:**

Our sample included a non‐Latino White population and Asian and Latino subpopulation groups using data from the 2013–2021 Medical Expenditure Panel Survey.

**Principal Findings:**

Asian and Latino subpopulations used health care services less frequently than the White population, with adjusted differences ranging from −3.2% points (95% CI: −3.9, −2.4) to −9.4 (−10.1, −8.7) for outpatient visits, −5.2 (−5.9, −4.5) to −12.4 (−15.2, −9.6) for office‐based provider visits, and −5.2 (−6.7, −3.8) to −19.1 (−21.6, −16.7) for prescription drug fills. Although certain low‐value services were reported less among Asian and Latino subpopulations, there were no differences in almost six out of twelve services when compared to the White population. These patterns were notable among Asian subpopulations (Indians, Chinese, Filipinos, and other Asians). Additionally, Asian and Latino subpopulation groups had distinct patterns in the use of low‐value care. Compared to the White population, Asian subpopulation groups had lower utilization of low‐value medications including benzodiazepines for depression (−11.5 [−15.1, −8.0] to −13.8 [−24.4, −3.3]) and opioids for back pain (−4.4 [−8.5, −0.3] to −10.1 [−13.6, −6.7]). Latino subpopulation groups had higher utilization of low‐value cervical cancer screening (5.7 [3.0–8.4] to 24.5 [16.9–32.1]) and lower utilization of magnetic resonance imaging/computed tomography for back pain (−1.6 [−2.4, −0.8] to −4.9 [−7.1, −2.6]) than the White population.

**Conclusions:**

Despite lower overall health care utilization, Asian and Latino subpopulations do not necessarily use the low‐value care examined in this study less than the White population. This suggests that lower overall health care utilization among Asian and Latino subpopulations may not solely be attributed to lower use of low‐value care.

**Summary**

- What is known on this topic
  ○ Studies have shown that Asians and Latinos use, on average, less health care, including preventive care, compared to the White population.
  ○ Our understanding of how Asian and Latino populations use low‐value services remains limited.
  ○ Asian and Latino subpopulation groups may experience significant variations in the utilization of low‐value care that are not well understood.
- What this study adds
  ○ All Asian and Latino subpopulation groups used health care services less frequently than the White population.
  ○ Some low‐value services were reported less among Asian and Latino subpopulations, but utilization for nearly six of the twelve low‐value services was similar to or higher than reported for the White population.
  ○ The results suggest that lower overall health care utilization among Asian and Latino subpopulations may not necessarily be caused by lower low‐value care utilization.

## 1 Introduction

Asian and Latino populations represent fast‐growing demographic groups within the United States (US) [1, 2]. In 2020, approximately 24 million Asian individuals and 62 million Latino individuals resided in the country, which collectively represent nearly a quarter of the general population [3]. This growth trend is projected to continue, with estimates indicating that by 2060, these numbers will increase to 36 million and 111 million, respectively, and together these populations will compose about 40% of the population. Research has found that Asian and Latino population groups use, on average, fewer health care services than the White population. For example, in 2016, the average per‐person health care spending was substantially lower among Asian and Latino individuals than among White individuals ($4692, $6025, and $8141, respectively) [4]. These disparities are partly attributable to unequal access to and use of primary care [5]. For example, the likelihood of receiving routine checkups was also lower among Asian and Latino individuals compared to White individuals [6].

Studies have primarily focused on measuring overall health care utilization or preventive care among Asian and Latino populations [7, 8, 9, 10, 11], which leaves a gap in our understanding of the utilization of low‐value care among Asian and Latino populations. Low‐value care, which is defined as services with minimal or no clinical benefit but incurring health care costs, is widespread in the US [12, 13, 14, 15]. Annual spending on low‐value care was estimated to range from $75.7 to $101.2 billion in 2019 [16]. Approximately 10% of US adults received low‐value treatments [15]. This phenomenon was more pronounced among vulnerable populations, including racial and ethnic minorities and individuals with lower income or education levels [17, 18, 19]. For example, Black individuals on Medicare receive more low‐value care than White individuals on Medicare [17, 20]. Although Asian and Latino populations are also considered minoritized populations, health care utilization patterns may differ from those of Black populations, which indicates that low‐value care use may not necessarily be high. However, evidence on the utilization of low‐value care within these groups remains limited.

While Asians and Latino populations are often treated as single monolithic groups, these populations are diverse in terms of culture, language, religion, immigration and migration patterns, histories in the US, and several other characteristics [1, 2]. With over 50 and 20 Asian and Latino subpopulation groups, respectively, each subpopulation group has unique socioeconomic and cultural characteristics that may influence health care access and utilization [7, 8, 9, 10, 11]. This suggests that substantial variations in the utilization of low‐value care may exist among different Asian and Latino subpopulation groups. Identifying these variations is critical for developing more targeted approaches tailored to the needs of each subpopulation. However, population‐based surveys often oversimplify by categorizing Asian and Latino populations into broad singular groups, which potentially mask the diversity within these populations [21]. As a result, less is known about the utilization of low‐value care among Asian and Latino subpopulations.

To address these knowledge gaps, we used a nationally representative US population survey for 2013–2021 and conducted two central analyses. First, we examined whether general health care utilization was lower among Asian and Latino subpopulation groups compared to the White population. Second, we investigated whether there were differences in the utilization of low‐value care among Asian and Latino subpopulation groups compared to the White population. Our analyses and model fitting were guided by the Andersen behavioral model of health care utilization, which is a widely used framework for understanding the factors influencing health care service use [22]. This model posits that health care utilization is shaped by three distinct determinants: predisposing, enabling, and need factors. By applying this framework, we aim to gain deeper insights into the complexities of health care utilization among Asian and Latino subpopulation groups.

## 2 Methods

### 2.1 Data

We analyzed repeated cross‐sectional data from the 2013 to 2021 Medical Expenditure Panel Survey (MEPS), which is a nationally representative survey of the US civilian non‐institutionalized population. The data are collected from interviews with individual households and their members and are augmented by data provided by hospitals, physicians, home healthcare providers, and pharmacies that have offered care to those same subjects and by their employers. MEPS collects data from two primary sources. The Household Component (HC) collects data from individual household members through survey questionnaires, and the Medical Provider Component (MPC) collects data from a sample of health care providers to MEPS HC respondents. The HC data include demographic, socioeconomic, and health characteristics. The MPC data include dates of visits or services, types of health care services used, and diagnoses codes for medical encounters. For this study, we used five datasets from MEPS: the full‐year consolidated data files from the HC, outpatient visits files, office‐based medical provider visit files, prescribed medicine files, and medical conditions files from the MPC. The analytic sample consisted of US adults (ages 18 years and older) with complete information.

The [redacted] Institutional Review Board deemed this study exempt because it used only publicly available data without patient identifiers. This study adhered to the STROBE reporting guidelines for cross‐sectional studies.

### 2.2 Outcomes

We included two types of outcome measures. First, we constructed binary measures of three general health care services: outpatient visits, office‐based medical provider visits, and prescription drug fills. These services were selected because they align directly with the settings in which we define low‐value care. Office‐based visits are appointments that occur primarily in office settings and clinics and do not include care received in hospitals, nursing homes, or patients' homes. In contrast, outpatient department visits take place in hospital outpatient departments, where patients access a variety of medical services. Second, we followed prior studies that used MEPS data [14, 23, 24, 25] and constructed binary measures of 12 low‐value services across the following three categories: (1) low‐value cancer screening (cervical [26], colorectal [27], and prostate cancer screening [28]); (2) low‐value medication use (antibiotic for acute upper respiratory infection [29, 30], antibiotic for influenza [29], use of benzodiazepine for depression [31], use of opioid for back pain [32], use of opioid for headache [33], and use of nonsteroidal anti‐inflammatory drug [NSAID] for individuals with hypertension, heart failure, or chronic kidney disease [32]); and (3) low‐value imaging tests (magnetic resonance imaging [MRI] or computed tomography [CT] for back pain [34], radiograph for back pain [34], and MRI or CT for headache [35]). Definitions outlining the criteria for each outcome measure are presented in Table 1. Additionally, we constructed a composite measure for the use of any low‐value service in each category. Previous studies have investigated approximately 40 low‐value services using claims data [12, 13, 36, 37]. However, due to limited measures in MEPS, we were unable to account for all low‐value services.

**TABLE 1:** Measures for low‐value care.

Measure	Source	MEPS data source	Measure (numerator)	Eligible population (denominator)
Cancer screening				
Cervical cancer screening	Vesco et al. [22]	Self‐report	Papanicolaou test in the given survey year	Women older than 65 years without a diagnosis of cervical cancer or other genital cancer found among women in the prior year
Colorectal cancer screening	Whitlock et al. [23]	Self‐report	Colonoscopy within past 10 years, sigmoidoscopy within past 5 years, or hemoccult test within past year	Individuals older than 75 years without a diagnosis of colon cancer in the prior year
Prostate cancer screening	Fenton et al. [24]	Self‐report	Prostate‐specific antigen test in the given survey year	Men older than 70 years without a diagnosis of prostate cancer in the prior year
Medication use				
Antibiotics for acute upper respiratory infection	Cooper et al. [25] Harris et al. [26]	Prescribed medicine	Antibiotic prescription during visit	Individuals with a primary diagnosis of acute upper respiratory infection without a diagnosis of bacterial infection, chronic obstructive pulmonary disease, or cancer in the given survey year (as competing diagnosis for acute upper respiratory infection)
Antibiotics for influenza	Cooper et al. [25]	Prescribed medicine	Antibiotic prescription during visit	Individuals with a primary diagnosis of influenza without a diagnosis of bacterial infection, chronic obstructive pulmonary disease, or cancer in the given survey year
Benzodiazepine for depression	Trangle et al. [27]	Prescribed medicine	Benzodiazepine prescription during visit	Individuals with a diagnosis of depression
Opioid for back pain	American Society of Neurology [28]	Prescribed medicine	Opioid prescription during visit	Individuals with a diagnosis of back pain but no diagnosis of fever or cancer in the give survey year
Opioid for headache	American Society of Anesthesiologists [29]	Prescribed medicine	Opioid prescription during visit	Individuals with a diagnosis of headache but no diagnosis of pregnancy, cancer, or epilepsy in the given survey year
NSAID use for hypertension, heart failure, or kidney disease	American Society of Anesthesiologists [29]	Prescribed medicine	NSAID prescription during visit	Individuals with a diagnosis of hypertension, hear failure, or kidney disease in the give survey year
Imaging				
MRI/CT for back pain	Chou et al. [30]	Office‐based and outpatient	MRI or CT scan during visit	Individuals with a diagnosis of back pain but no diagnosis of fever or cancer in the given survey year
Radiograph for back pain	Chou et al. [30]	Office‐based and outpatient	Radiograph during visit	Individuals with a diagnosis of back pain but no diagnosis of fever or cancer in the given survey year
MRI/CT for headache	American College of Radiology [31]	Office‐based and outpatient	MRI or CT scan during visit	Individuals with a diagnosis of headache but no diagnosis of pregnancy, cancer, or epilepsy in the given survey year

Abbreviations: CT, computed tomography; MRI, magnetic resonance imaging; NSAID, nonsteroidal anti‐inflammatory drug.

For each low‐value care measure, we identified individuals who were eligible for the measure (the denominator) based on age, sex, and health conditions based on *the International Classification of Diseases, Ninth Revision, Clinical Modification* (ICD‐9‐CM) or *the International Classification of Diseases, Tenth Revision, Clinical Modification* (ICD‐10‐CM). Subsequently, we determined whether these eligible individuals received specific low‐value services (the numerator). The sample size differed for each outcome measure.

### 2.3 Key Independent Variables

Our primary independent variable was self‐reported race and ethnicity. Participants were asked to provide information about their race and ethnicity based on categories listed in the questionnaire. We categorized individuals into 12 mutually exclusive population and subpopulation groups: non‐Latino “White” individuals, non‐Latino “Asian” individuals (Asian Indian, Chinese, Filipino, and Other Asian), and “Latino” individuals (Central/South American, Cuban, Dominican, Mexican, Other Latino, and Puerto Rican). The categories of “other Asian” or “other Latino” include those who self‐identified as Asian or Latino but not as any of the specified subpopulation groups. Black individuals and individuals of other races and ethnic groups were excluded from our analyses. We selected White individuals as the reference, as this population has been shown to have greater health care use compared to minoritized groups [4, 5, 6, 7, 8, 9, 10, 11].

### 2.4 Covariates

We used the Andersen behavioral model of health care utilization to guide covariate selection for statistical modeling [22]. The model posits that health care use is determined by predisposing, enabling, and need factors. Thus, we categorized the individual‐level characteristics into three categories: predisposing (age and sex), enabling (employment status, marital status, education, family income, and health insurance), and need factors (number of chronic conditions from a total 20 conditions).

### 2.5 Statistical Analyses

We calculated sample characteristics by race and ethnicity. We then estimated eligible sample sizes and unadjusted proportions for each low‐value care measure within these racial and ethnic categories. To quantify differences in the utilization of both general health care services and low‐value services across these groups, we ran a linear probability model after controlling for individual‐level characteristics and year‐fixed effects as follows:

$$Y_{\mathrm{it}}=\alpha +\text{Race}/{\text{ethnicity}}_{\mathrm{it}}+X_{\mathrm{it}}\gamma +V_{t}\eta +\varepsilon_{\mathrm{it}}$$

where i indexes individuals, t indexes year, and Y represents an outcome measure for individual i at year t. Race/ethnicity is an indicator of mutually exclusive self‐reported racial and ethnic subpopulation groups. X is a vector containing the predisposing, enabling, and need factors described earlier. V is a year‐fixed effect. ε is an error term.

Using the marginal effects from these models, we estimated the mean adjusted values of the outcomes for each racial and ethnic group and subpopulation group while holding all other variables constant except the variable of interest. Furthermore, we estimated the adjusted differences in outcomes among Asian and Latino subpopulations relative to the White population. Recognizing potential variations in the association of individual‐level characteristics with outcomes among Asian and Latino population groups, we conducted separate analyses for each group. These estimates represent differences in outcomes, assuming that individuals in each racial and ethnic group have, on average, the same characteristics included as the covariates—except for race and ethnicity—highlighting the differences attributable to race and ethnicity. This approach facilitates clearer comparisons but may underestimate existing disparities, as factors like income and employment status were also accounted for in the adjustment process. The Institute of Medicine defines disparities as differences in utilization influenced by various factors beyond health status and personal preferences, including socioeconomic and environmental factors [38]. To address this limitation, we performed sensitivity analyses by only adjusting for predisposing factors (age and sex) and need factors (number of chronic conditions). As a result, the former estimates can be interpreted as the lower bound of racial and ethnic disparities, while the latter estimates represent the upper bound.

For all analyses, we used survey weights to generate nationally representative estimates. Since the MEPS sample was selected using various survey designs, including stratifications, clustering, multiple stages of selection, and disproportionate sampling, we accounted for this complex survey design in our standard error estimation by using MEPS survey weights to produce estimates and employing an appropriate technique to derive standard errors associated with these weighted estimates. This approach helps ensure that our standard error calculations accurately reflect the true uncertainty in our results. Data were analyzed using Stata statistical software version 16.1 (StataCorp).

## 3 Results

### 3.1 Sample Characteristics

The unweighted sample consisted of 153,379 individuals (mean [SD] age, 48.4 [18.0]; 51.3 female). Racial and ethnic population groups included 96,013 White, 2849 Asian Indian, 2554 Chinese, 2047 Filipino, 4681 other Asian, 7160 Central/South American, 2256 Cuban, 1732 Dominican, 27,845 Mexican, 2542 other Latino, and 3700 Puerto Rican individuals. Sample characteristics and unadjusted outcomes are presented in Appendix Table A and Table 2, respectively.

**TABLE 2:** Unadjusted estimates of low‐value care use among Asian and Latino subpopulations.

Outcome	White		Asian Indian		Chinese		Filipino		Other Asian	
	Eligible sample, *N*	% of receipt	Eligible sample, *N*	% of receipt	Eligible sample, *N*	% of receipt	Eligible sample, *N*	% of receipt	Eligible sample, *N*	% of receipt
Cancer screening										
Cervical cancer screening	4276	18.3	49	26.5	116	18.1	102	16.7	238	15.5
Colorectal cancer screening	3345	3.6	23	4.3	82	0.0	61	3.3	169	5.9
Prostate cancer screening	2274	56.8	32	46.9	65	58.5	41	46.3	100	36.0
Medication use										
Antibiotics for acute upper respiratory infection	9065	31.0	267	17.6	192	16.1	163	16.0	408	16.4
Antibiotics for influenza	3883	13.2	151	17.9	139	12.2	89	13.5	177	13.6
Benzodiazepine for depression	11,796	25.4	58	10.3	58	8.6	55	9.1	176	13.1
Opioid for back pain	10,696	17.6	155	5.8	145	4.8	122	6.6	282	8.5
Opioid for headache	4370	3.4	76	0.0	46	0.0	53	0.0	150	6.0
NSAID use for hypertension, heart failure, or kidney disease	10,772	10.6	184	8.7	232	6.5	285	7.7	522	7.5
Imaging use										
MRI/CT for back pain	10,696	8.8	155	7.1	145	9.7	122	6.6	282	7.1
Radiograph for back pain	10,696	13.0	155	14.2	145	8.3	122	11.5	282	16.0
MRI/CT for headache	4370	5.1	76	3.9	46	15.2	53	3.8	150	4.7

Outcome	Central/South American		Cuban		Dominican		Mexican		Other Hispanic		Puerto Rican	
	Eligible sample, *N*	% of receipt	Eligible sample, *N*	% of receipt	Eligible sample, *N*	% of receipt	Eligible sample, *N*	% of receipt	Eligible sample, *N*	% of receipt	Eligible sample, *N*	% of receipt
Cancer screening												
Cervical cancer screening	206	34.5	116	37.9	74	24.3	540	20.7	61	19.7	123	34.1
Colorectal cancer screening	93	3.2	105	5.7	49	4.1	352	3.7	47	4.3	64	7.8
Prostate cancer screening	52	59.6	77	55.8	25	60.0	265	39.6	29	41.4	44	50.0
Medication use												
Antibiotics for acute upper respiratory infection	512	16.8	139	19.4	111	18.0	1947	21.6	196	29.6	351	21.7
Antibiotics for influenza	241	19.1	78	20.5	65	13.8	1086	12.9	97	12.4	202	12.4
Benzodiazepine for depression	311	17.0	185	45.4	109	23.9	1281	19.8	210	20.0	520	29.2
Opioid for back pain	440	11.6	147	9.5	160	13.1	1455	13.4	219	17.4	463	20.3
Opioid for headache	270	1.5	57	5.3	101	5.0	921	2.9	90	1.1	229	2.2
NSAID use for hypertension, heart failure, or kidney disease	512	16.8	139	19.4	111	18.0	1947	21.6	196	29.6	351	21.7
Imaging use												
MRI/CT for back pain	440	6.4	147	10.9	160	5.0	1455	4.5	219	4.1	463	6.7
Radiograph for back pain	440	14.3	147	12.2	160	14.4	1455	11.9	219	11.4	463	13.4
MRI/CT for headache	270	4.8	57	7.0	101	5.9	921	4.0	90	4.4	229	5.7

*Note:* Survey weights were used to generate nationally representative estimates.

Abbreviations: CT, computed tomography; MRI, magnetic resonance imaging; NSAID, nonsteroidal anti‐inflammatory drug.

### 3.2 Health Care Utilization

**FIGURE 1:**
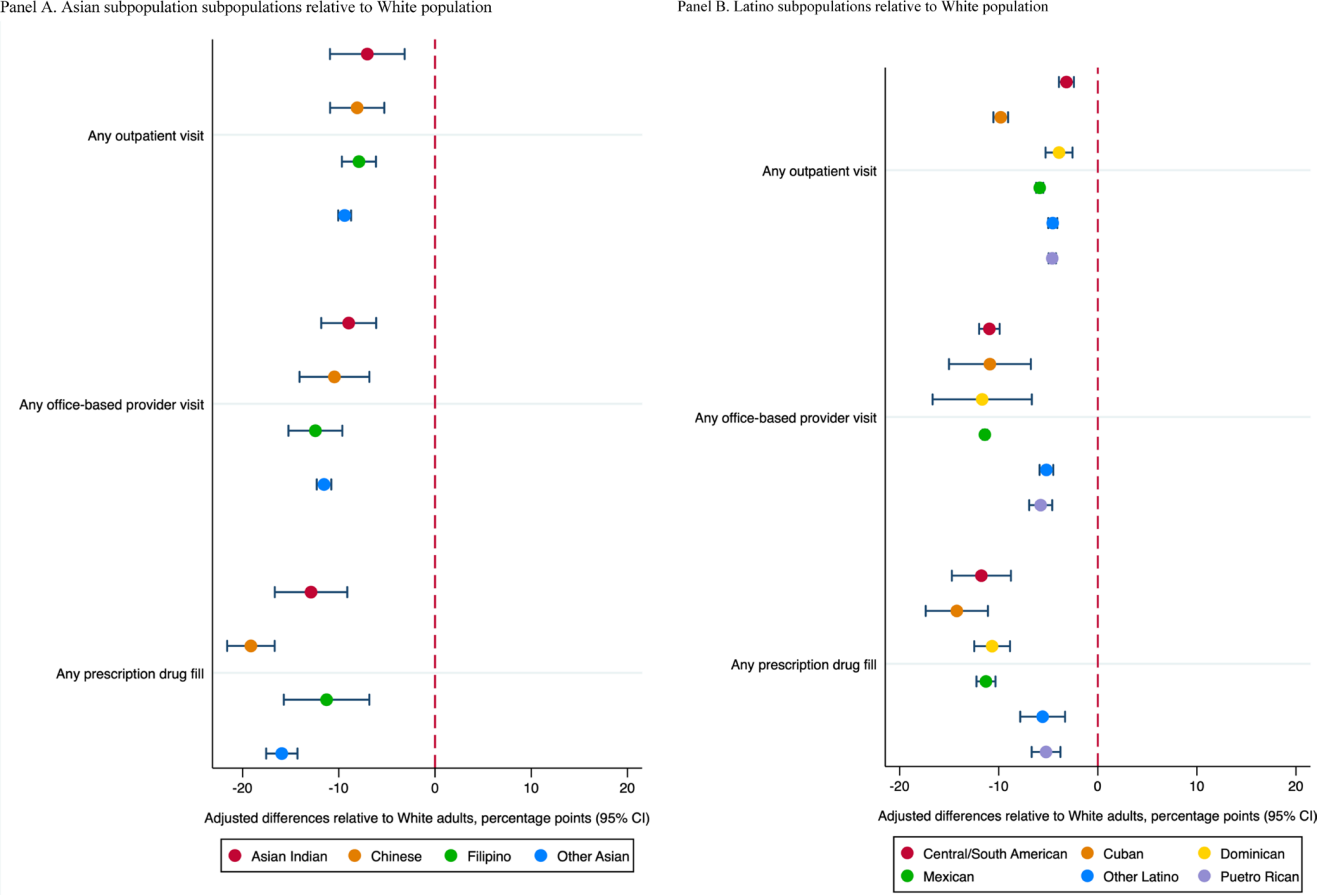
Adjusted differences in outpatient visit, office‐based provider visit, and prescription drug fill among Asian and Latino subpopulations relative to White population. Panel (A) Asian subpopulations relative to the White population. Panel (B) Latino subpopulations relative to White population. To quantify differences in the utilization of general health care services across Asian and Latino subpopulation groups, we ran a linear probability model after controlling for individual‐level characteristics and year‐fixed effects. Using the marginal effects from these models, we estimated the mean adjusted values of the outcomes for each racial and ethnic subpopulation group while holding all other variables constant except the variable of interest. Furthermore, we estimated the adjusted differences in outcomes among Asian and Latino subpopulation groups relative to the White population.

Compared to the White population, all Asian and Latino subpopulation groups had lower utilization of general health care services, with more pronounced differences observed among Asian subpopulation groups. Specifically, the likelihood of having outpatient visits, office‐based provider visits, and prescription drug fills among Asian subpopulation groups relative to the White population was lower by −7.0% points (95% CI: −10.9, −3.2) for Asian Indian individuals to −9.4% points (95% CI: −10.1, −8.7) for other Asian individuals, −9.0% points (95% CI: −11.8, −6.1) for Asian Indian individuals to −12.4% points (95% CI: −15.2, −9.6) for Filipino individuals, and −11.3% points (95% CI: −15.7, −6.8) for Filipino individuals to −19.1% points (95% CI: −21.6, −16.7) for Chinese individuals, respectively (Figure 1 Panel A). The likelihood of having outpatient visits, office‐based provider visits, and prescription drug fills among Latino subpopulation groups relative to the White population was lower by −3.2% points (95% CI: −3.9, −2.4) for Central/South American individuals to −9.8% points (95% CI: −10.5, −9.1) for Cuban individuals, −5.2% points (95% CI: −5.9, −4.5) for other Latino individuals to −11.7% points (95% CI: −16.7, −6.7) for Dominican individuals, and −5.2% points (95% CI: −6.7, −3.8) for Puerto Rican individuals to −14.2 (95% CI: −17.4, −11.1) for Cuban individuals, respectively (Figure 1 Panel B).

### 3.3 Low‐Value Care

**FIGURE 2:**
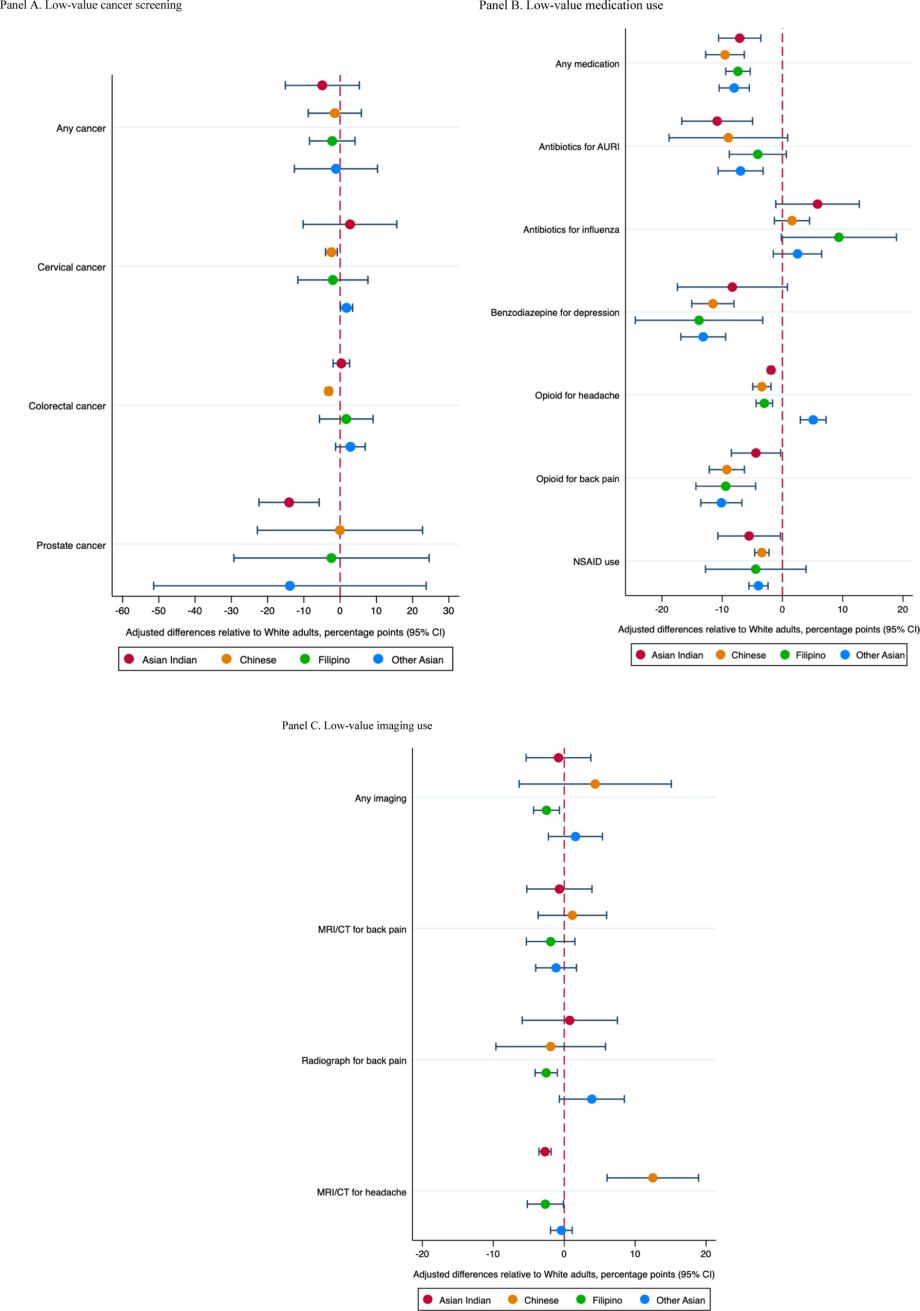
Adjusted differences in use of low‐value care among Asian subpopulation groups relative to White population. Panel (A) Low‐value cancer screening. Panel (B) Low‐value medication use. Panel (C) Low‐value imaging use. To quantify differences in the utilization of low‐value care services across Asian subpopulation groups, we ran a linear probability model after controlling for individual‐level characteristics and year‐fixed effects. Using the marginal effects from these models, we estimated the mean adjusted values of the outcomes for each Asian subpopulation group while holding all other variables constant except for the variable of interest. Furthermore, we estimated the adjusted differences in outcomes among Asian subpopulation groups relative to the White population.

**FIGURE 3:**
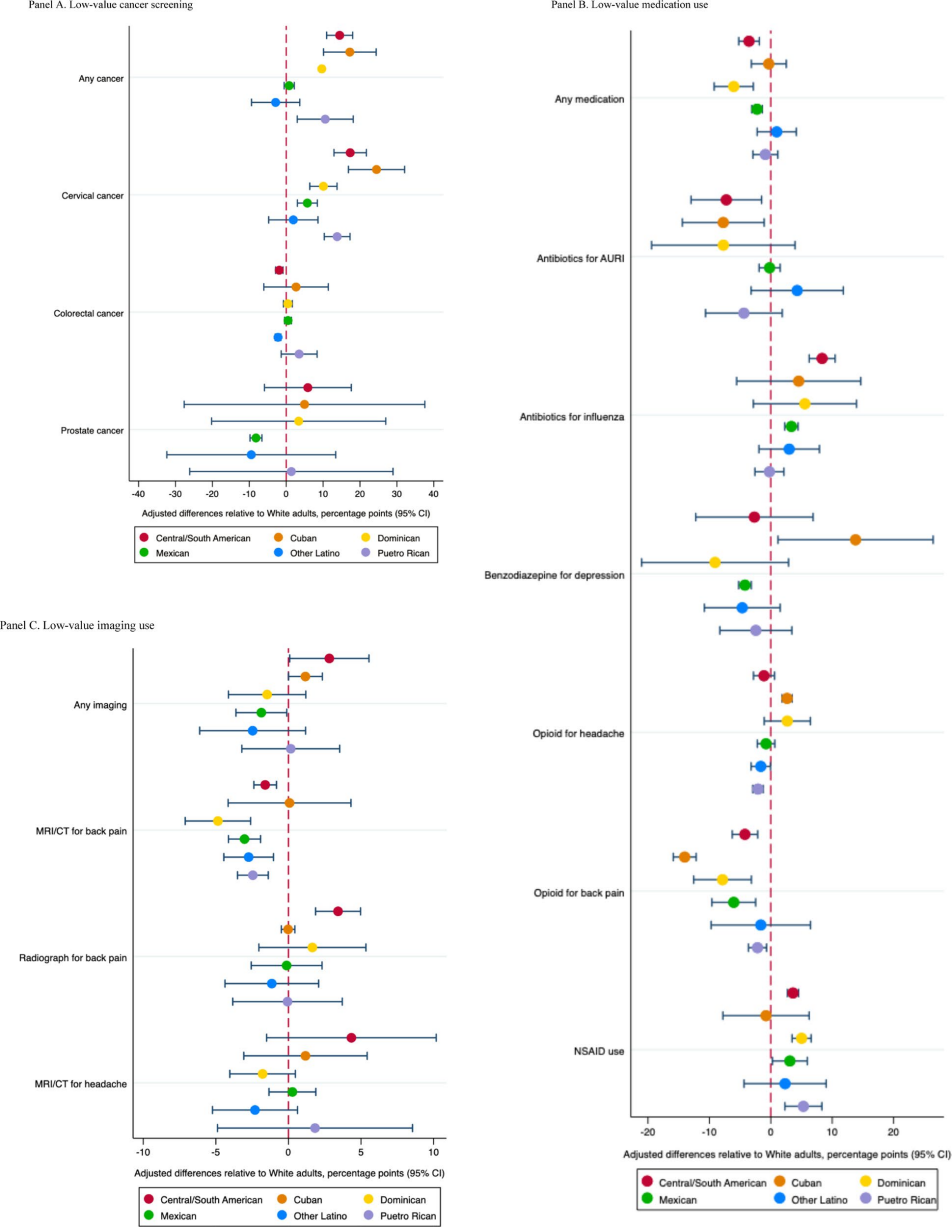
Adjusted differences in the use of low‐value care among Latino subpopulation groups relative to the White population. Panel (A) Low‐value cancer screening. Panel (B) Low‐value medication use. Panel (C) Low‐value imaging use. To quantify differences in the utilization of low‐value care services across Latino subpopulation groups, we ran a linear probability model after controlling for individual‐level characteristics and year fixed effects. Using the marginal effects from these models, we estimated the mean adjusted values of the outcomes for each Latino subpopulation group while holding all other variables constant except the variable of interest. Furthermore, we estimated the adjusted differences in outcomes among Latino subpopulation groups relative to the White population.

While certain low‐value services were used less among Asian and Latino subpopulation groups, utilization rates for more than half of the low‐value services examined were similar to or even higher than those observed in the White population. Among Asian subpopulation groups, of 12 low‐value services, significantly lower utilization of low‐value care was observed in five services for Asian Indian, seven for Chinese, six for Filipino, and four for other Asian individuals, respectively (Figure 2 Panels A, B, and C and Appendix Table B). Among Latino subpopulation groups, of 12 low‐value services, significantly lower utilization of low‐value care was observed in six services for Central/South American, three for Cuban, two for Dominican, five for Mexican, two for other Latino, and four for Puerto Rican individuals (Figure 3 Panels A, B, and C and Appendix Table C). The majority of the differences were observed in low‐value medication use. In the sensitivity analysis, we found that, while enabling factors contributed to reducing differences, the results remained relatively consistent (Appendix Tables D and E).

While notable differences were observed in some low‐value services, the patterns of use of low‐value care differed within Asian and Latino subpopulation groups. Among Asian subpopulation groups, the use of low‐value care was more pronounced in low‐value medications: antibiotics for acute upper respiratory infection (ranging from −6.9% points [95% CI: −10.7, −3.2] for other Asian individuals to −10.8% points [95% CI: −16.7, −5.0] for Asian Indian individuals), benzodiazepines for depression (ranging from −11.5% points [95% CI: −15.1, −8.0] for Chinese individuals to −13.8% points [95% CI: −24.4, −3.3] for Filipino individuals), opioids for back pain (ranging from −4.4% points [95% CI: −8.5, −0.3] for Asian Indian individuals to −10.1% points [95% CI: −13.6, −6.7] for other Asian individuals), opioids for headache (ranging from −1.9% points [95% CI: −2.4, −1.4] for Asian Indian individuals to −3.4% points [95% CI: −4.9, −1.9] for Chinese individuals), and NSAID use for hypertension, heart failure, or kidney disease (ranging from −5.1% points [95% CI: −7.1, −3.1] for Chinese individuals to −14.6% points [95% CI: −18.4, −10.8] for Other Asian individuals) (Figure 2 Panels A, B, and C and Appendix Table B). Additionally, significantly lower utilization was observed in some cancer screening and imaging use: −2.4% points (95% CI: −4, −0.8) for cervical cancer screening among Chinese individuals, −3.2% points (95% CI: −4.0, −2.3) for colorectal cancer screening among Chinese individuals, −14.1% points (95% CI: −22.4, −5.8) for prostate cancer screening among Asian Indian individuals, −2.5% points (95% CI: −4.1, −1) for radiographs for back pain among Filipino individuals, and −2.7% points (95% CI: −3.6, −1.8) and −2.7% points (95% CI: −5.2, −0.1) for MRI/CT for headache among Asian Indian and Filipino individuals.

Latino subpopulation groups had similar patterns of lower utilization of low‐value medications as Asian subpopulation groups; however, the overall difference was less pronounced: antibiotics for acute upper respiratory infection (ranging from −7.2% points [95% CI: −13.0, −1.5] for Central/South American individuals to −7.7% points [95% CI: −14.4, −1.1] for Cuban individuals), benzodiazepines for depression (−4.2% points [95% CI: −5.2, −3.2] for Mexican individuals), opioids for back pain (−2.3% points [95% CI: −3.2, −1.4] for Mexican individuals), opioids for headache (ranging from −2.2% points [95% CI: −3.6, −0.7] for Puerto Rican individuals to −14.0% points [95% CI: −15.9, −12.2] for Cuban individuals), NSAID use for hypertension, heart failure, or kidney disease (ranging from −4.2% points [95% CI: −5.8, −2.6] for Puerto Rican individuals to −9.3% points [95% CI: −13.7, −5.0] for Cuban individuals) (Figure 3 Panels A, B, and C and Appendix Table C). Lower utilization in imaging services was also evident: MRI/CT for back pain (ranging from −3.3% points [95% CI: −5.9, −0.8] for Central/South American individuals to −5.5% points [95% CI: −8.4, −2.5] for Mexican individuals), and radiographs for back pain (ranging from −1.6% points [95% CI: −2.4, −0.8] for Central/South American individuals to −4.9% points [95% CI: −7.1, −2.6] for Dominican individuals).

Some instances of low‐value care were more frequently observed among Asian and Latino subpopulation groups compared to the White population. Notably, higher utilization of low‐value cancer screening was observed among Latino subpopulation groups. Cervical cancer screening was more prevalent among other Asian, Central/South American, Cuban, Dominican, and Puerto Rican individuals, with increases of 1.8% points (95% CI: 0.1, 3.4), 14.5% points (95% CI: 11.0, 18.0), 17.2% points (95% CI: 10.1, 24.4), 9.6% points (95% CI: 9.1, 10.2), and 10.6% points (95% CI: 3.0, 18.2). However, there were no or small differences in colorectal and prostate cancer screening. Additionally, there were differences observed in the use of low‐value medication and imaging, albeit they were modest. Antibiotics for influenza were more frequently observed among Central/South American and Mexican individuals, with increases of 8.4% points (95% CI: 6.3, 10.5) and 3.4% points (95% CI: 2.3, 4.4). Benzodiazepine use for depression and opioids for headache was higher among Cuban individuals, with increases of 13.8% points (95% CI: 1.2, 26.4) and 2.7% points (95% CI: 1.8, 3.5). Additionally, opioid use for headache was higher among other Asian individuals, with an increase of 5.1% points (95% CI: 3.0, 7.3), and MRI/CT for headache was more common among Chinese and Central/South American individuals, with increases of 12.5% points (95% CI: 6.0, 18.9) and 3.4% points (95% CI: 1.9, 5.0).

## 4 Discussion

In this nationally representative study, we observed that Asian and Latino subpopulation groups used fewer health care services than the White population, which is consistent with prior findings [7, 8, 9, 10, 11]. However, we also discovered that these groups used approximately six out of 12 low‐value services similarly to the White population. These findings indicate that the lower overall health care utilization among Asian and Latino subpopulations may not solely be attributed to the use of low‐value care. Studies have identified significant barriers to accessing health care among Asian and Latino populations, such as language and cultural barriers, limited health literacy, lack of health insurance, and immigration status [39, 40, 41]. Nonetheless, our findings suggest that these factors may not fully explain differences in the use of low‐value care. Other studies have found that these groups use preventive services at lower levels than the White population [8, 11], which potentially contributes to lower health care utilization. Taken together, the utilization patterns among Asian and Latino subpopulation groups were observed to differ between high‐ and low‐value care, which aligns with previous research on immigrant populations [42].

Moreover, our study found variations in low‐value care utilization within Asian and Latino subpopulation groups. One notable finding was the consistent lower utilization of low‐value medications among Asian subpopulation groups. This observation could be partly explained by lower prescription drug use among Asian subpopulation groups [43], particularly pain medications, which may include a reduced use of low‐value pain medications. Conversely, Latino subpopulation groups had higher utilization of low‐value cancer screenings than the White population. This result could be attributed to high enrollment rates in managed care plans among Latino subpopulation groups [44], which typically encourage the uptake of preventive services. However, certain cancer screenings provide significant benefits for some age groups while providing limited value for others. Indeed, screening for cervical, colorectal, and prostate cancers has been deemed low‐value for elderly adults [26, 27, 28]. As individuals often lack awareness of the distinction between high‐value and low‐value screenings, greater access to high‐value care might inadvertently lead to greater use of low‐value care [17]. Understanding the underlying mechanisms behind this observation is important. Although we could not fully investigate these mechanisms, our findings indicate that individual‐level factors have only a minor impact on the use of low‐value care, which suggests that provider‐related factors may be more influential.

Our findings should be interpreted with caution. To the best of our knowledge, there is a limited literature providing a comprehensive list of low‐value services. As a result, it is difficult to quantify the proportion of the 12 services we analyzed within the broader category of low‐value services or to determine the extent to which the overall lower health care utilization observed among Asian and Latino populations compared to Whites is attributable to differences in low‐value service use. However, several institutions or organizations have attempted to define and estimate low‐value care. For example, the MedInsight Health Waste Calculator examined more than 48 low‐value services using claims data. Additionally, recent studies have examined 26, 32, 40, and 41 measures, respectively [12, 13, 36, 37]. This suggests that our analyses account for only about 25% of the measurable low‐value care documented in the literature. Due to the limited availability of measures in MEPS, we could not account for all low‐value services, indicating that Asian and Latino populations may have used even fewer low‐value services not included in our study. Therefore, further research is needed to determine whether these findings are consistently observed in other contexts of low‐value care.

Our findings underscore the need for a multifaceted approach to address low‐value care utilization. Prior research has highlighted that the utilization of low‐value care is influenced by both patient and provider factors. Thus, interventions consisting of multiple components targeting both patient and provider roles hold the most promise for decreasing the use of low‐value care [45]. Specifically, public awareness initiatives, such as the “Choosing Wisely” campaign and “U.S. Antibiotic Awareness Week,” are essential for addressing misconceptions about low‐value services and promoting informed decision‐making [46]. Provider training based on evidence‐based guidelines can further support efforts to minimize the use of low‐value care. Additionally, integrating technology‐based tools into clinical workflows can aid providers in making evidence‐based decisions at the point of care. For instance, electronic health records with built‐in clinical decision support tools can assist providers in identifying and avoiding low‐value services [47]. Moreover, transitioning to value‐based care reimbursement models, such as the Accountable Care Organization models, can address the systemic factors driving low‐value care utilization [48]. The Centers for Medicare and Medicaid Services aims to enroll all Medicare beneficiaries and most Medicaid beneficiaries enrolled in accountable, value‐based care programs by 2030 [49], underscoring the urgent need for effective policy development to achieve these goals.

These approaches, however, may have varying impacts among Asian and Latino subpopulation groups. Improving general awareness about low‐value practices may decrease the use of low‐value care [46], but Asian and Latino subpopulation groups have lower awareness, which could potentially lead to higher utilization of low‐value care. Therefore, it is essential to develop culturally competent strategies with language supports to discourage low‐value care in these populations. Moreover, our study highlights that the utilization pattern of low‐value care varied considerably across different measures and racial/ethnic groups, suggesting a singular corrective intervention is unlikely to be universally effective. Indeed, each subpopulation group has unique socioeconomic and cultural characteristics that may influence health care utilization [7, 8, 9, 10, 11]. Nonetheless, there is limited understanding of the mechanisms that drive the use of low‐value care among Asian and Latino subpopulation groups. Thus, further research is needed to explore the underlying mechanisms within each specific subpopulation, which will inform the development of targeted policy and practice strategies.

### 4.1 Limitations

First, our study focused on specific low‐value care services, and, thus, our findings may not be applicable to other low‐value care services. Second, as our measures of low‐value care were self‐reported, there is a possibility of reporting errors. In particular, low‐value care may be underreported among minoritized populations, such as Asian and Latino groups, due to inconsistent data collection. Third, our ability to identify all relevant exclusions for low‐value care measurement was constrained. MEPS reports health conditions based on 3‐digit *ICD‐9‐CM* or *ICD‐10‐CM* diagnosis and procedure codes, which may limit the ability to identify competing diagnoses or exclude conditions associated with clinical red flags. Consequently, misclassification of individuals and conditions may have occurred in our measurement of low‐value care. Fourth, we did not distinguish Asian and Latino subpopulation groups by citizenship or documentation statuses, as MEPS does not provide measures on these demographics. Fifth, our study period included the COVID‐19 pandemic, which could impact our findings. Further research is warranted to explore the pandemic's effect on the utilization of low‐value care among different racial and ethnic groups. Finally, while we adjusted for differences in sample characteristics, residual differences in individual‐level characteristics may have persisted. For instance, English language proficiency may be a primary factor influencing health care utilization. However, due to limited measures in MEPS, we were unable to include all relevant factors in our study.

## 5 Conclusions

Despite low overall health care utilization, Asian and Latino subpopulation groups do not necessarily use low‐value care less frequently than the White population. This suggests that the lower overall health care utilization patterns among Asian and Latino subpopulation groups cannot solely be attributed to reduced use of low‐value care. Therefore, simply improving health care access may not be sufficient. Additionally, vulnerable populations face barriers to accessing health services, yet they often rely on low‐value care. This dual challenge underscores the complexity of addressing inequities in health care utilization among these groups.

## Conflicts of Interest

The authors declare no conflicts of interest.

## Supporting information


**Data S1.** Supporting Information.
