# Supplementary material for: Does Low‐Value Care Explain Health Care Utilization Inequities Among Asian and Latino Populations?
Source: Health Serv Res. 2025 Mar 20;60(4):e14610. doi: 10.1111/1475-6773.14610 (PMC12277110; doi:10.1111/1475-6773.14610)
Supplement: Supplementary file 1 — Data S1. Supporting Information. [file HESR-60-0-s001.docx]

**APPENDIX**

Appendix Table A. Sample characteristics by race and ethnicity.

Appendix Table B. Adjusted differences in use of low-value care among Asian subpopulation groups relative to White population (after adjusting for all factors).

Appendix Table C. Adjusted differences in use of low-value care among Latino subpopulation groups relative to White population (after adjusting for all factors).

Appendix Table D. Adjusted differences in use of low-value care among Asian subpopulation groups relative to White population (after adjusting for age, sex, and chronic conditions).

Appendix Table E. Adjusted differences in use of low-value care among Latino subpopulation groups relative to White population (after adjusting for age, sex, and chronic conditions).

Appendix Table A. Sample characteristics by race and ethnicity.

|  | Weighted % | | | | | | | | | |
| --- | --- | --- | --- | --- | --- | --- | --- | --- | --- | --- |
| Characteristics | White | Asian Indian | Chinese | Filipino | Central/South American | Cuban | Dominican | Mexican | Other Hispanic | Puerto Rican |
| Age |  |  |  |  |  |  |  |  |  |  |
| 18-24 | 8.5 | 8.7 | 10.3 | 10.6 | 10.2 | 12.7 | 9.3 | 14.0 | 15.5 | 16.8 |
| 25-44 | 16.0 | 25.5 | 22.5 | 16.8 | 20.0 | 22.5 | 18.6 | 22.3 | 24.3 | 21.7 |
| 45-64 | 14.9 | 26.1 | 18.3 | 18.8 | 18.9 | 23.7 | 13.8 | 17.5 | 22.1 | 18.9 |
| 65-74 | 16.5 | 18.1 | 18.2 | 18.8 | 19.2 | 19.4 | 19.1 | 17.3 | 17.6 | 18.0 |
| 75+ | 18.8 | 10.5 | 13.7 | 16.5 | 13.6 | 12.3 | 16.1 | 13.4 | 11.0 | 10.3 |
| Female | 51.4 | 49.6 | 55.3 | 59.3 | 54.2 | 52.8 | 50.6 | 55.1 | 49.0 | 51.9 |
| Employed | 62.7 | 68.6 | 64.4 | 64.9 | 59.7 | 71.7 | 63.5 | 58.1 | 66.6 | 63.9 |
| Married | 57.2 | 73.0 | 64.8 | 59.3 | 57.7 | 51.5 | 44.9 | 42.2 | 49.5 | 42.3 |
| Education |  |  |  |  |  |  |  |  |  |  |
| High school or lower | 6.7 | 7.3 | 8.2 | 5.9 | 12.2 | 25.3 | 17.8 | 29.4 | 33.1 | 12.6 |
| College graduate | 40.7 | 16.1 | 23.7 | 32.8 | 36.3 | 39.5 | 41.7 | 41.1 | 43.9 | 47.1 |
| Advanced degree | 52.6 | 76.6 | 68.1 | 61.3 | 51.5 | 35.2 | 40.4 | 29.5 | 23.0 | 40.4 |
| Family income |  |  |  |  |  |  |  |  |  |  |
| <200% of FPL | 21.6 | 15.9 | 20.7 | 17.9 | 30.4 | 33.1 | 39.5 | 50.3 | 42.3 | 28.2 |
| 200-399% of FPL | 27.5 | 19.0 | 18.1 | 26.7 | 26.9 | 37.1 | 30.8 | 30.1 | 33.5 | 31.5 |
| ≥400% of FPL | 50.9 | 65.1 | 61.2 | 55.4 | 42.6 | 29.8 | 29.7 | 19.6 | 24.2 | 40.4 |
| Health insurance coverage |  |  |  |  |  |  |  |  |  |  |
| Any coverage | 94.5 | 95.6 | 95.7 | 92.7 | 91.9 | 75.9 | 81.8 | 85.0 | 73.8 | 87.2 |
| Medicaid coverage | 8.4 | 11.6 | 12.2 | 10.5 | 18.4 | 14.5 | 15.5 | 39.9 | 17.5 | 14.3 |
| Medicare coverage | 27.6 | 9.3 | 16.1 | 17.7 | 18.5 | 9.0 | 25.2 | 16.0 | 10.0 | 15.2 |
| Private coverage | 60.7 | 77.0 | 70.9 | 68.0 | 60.4 | 55.5 | 49.5 | 39.3 | 49.2 | 59.9 |
| US census region |  |  |  |  |  |  |  |  |  |  |
| Northeast | 19.1 | 33.2 | 23.7 | 11.1 | 14.3 | 22.0 | 7.6 | 72.6 | 2.4 | 17.3 |
| Midwest | 25.7 | 14.7 | 10.3 | 8.9 | 12.1 | 5.3 | 1.8 | 1.8 | 10.8 | 8.0 |
| South | 35.1 | 27.3 | 16.5 | 12.7 | 26.1 | 47.4 | 86.9 | 23.9 | 33.9 | 33.6 |
| West | 20.2 | 24.8 | 49.5 | 67.4 | 47.5 | 25.3 | 3.7 | 1.6 | 52.9 | 41.1 |
| Number of chronic conditions |  |  |  |  |  |  |  |  |  |  |
| 0 | 17.7 | 5.5 | 8.1 | 6.1 | 8.0 | 7.7 | 12.4 | 10.3 | 7.5 | 12.1 |
| 1-2 | 15.5 | 8.4 | 6.2 | 13.2 | 10.3 | 8.1 | 12.8 | 10.5 | 9.8 | 11.2 |
| 3-5 | 3.4 | 0.9 | 0.2 | 1.2 | 1.0 | 1.1 | 2.3 | 1.7 | 1.2 | 2.0 |
| 6+ | 0.2 | 0.0 | 0.0 | 0.0 | 0.1 | 0.0 | 0.0 | 0.0 | 0.1 | 0.1 |

Appendix Table B. Adjusted differences in use of low-value care among Asian subpopulation groups relative to White population (after adjusting for all factors).

|  | Adjusted differences (relative to White adults), percentage points (95% CI) | | | |
| --- | --- | --- | --- | --- |
| Outcome | Asian Indian | Chinese | Filipino | Other Asian |
| Cancer screening | -4.9 (-15.1, 5.3) | -1.5 (-8.8, 5.8) | -2.2 (-8.5, 4.1) | -1.1 (-12.6, 10.3) |
| Cervical cancer screening | 2.7 (-10.2, 15.6) | -2.4 (-4, -0.8) | -2 (-11.7, 7.7) | 1.8 (0.1, 3.4) |
| Colorectal cancer screening | 0.4 (-1.9, 2.6) | -3.2 (-4, -2.3) | 1.7 (-5.7, 9.1) | 2.8 (-1.2, 6.9) |
| Prostate cancer screening | -14.1 (-22.4, -5.8) | 0 (-22.8, 22.7) | -2.4 (-29.3, 24.5) | -13.8 (-51.4, 23.7) |
| Medication use | -11.5 (-14.7, -8.3) | -5.1 (-7.1, -3.1) | -9.3 (-13, -5.6) | -14.6 (-18.4, -10.8) |
| Antibiotics for acute upper respiratory infection | -10.8 (-16.7, -5) | -9 (-18.8, 0.9) | -4.1 (-8.8, 0.6) | -6.9 (-10.7, -3.2) |
| Antibiotics for influenza | 5.8 (-1.1, 12.8) | 1.6 (-1.3, 4.5) | 9.4 (-0.2, 18.9) | 2.5 (-1.5, 6.5) |
| Benzodiazepine for depression | -8.3 (-17.4, 0.8) | -11.5 (-15.1, -8) | -13.8 (-24.4, -3.3) | -13.1 (-16.9, -9.4) |
| Opioid for back pain | -4.4 (-8.5, -0.3) | -9.2 (-12.2, -6.3) | -9.4 (-14.4, -4.5) | -10.1 (-13.6, -6.7) |
| Opioid for headache | -1.9 (-2.4, -1.4) | -3.4 (-4.9, -1.9) | -3 (-4.4, -1.6) | 5.1 (3, 7.2) |
| NSAID use for hypertension, heart failure, or kidney disease | -11.5 (-14.7, -8.3) | -5.1 (-7.1, -3.1) | -9.3 (-13, -5.6) | -14.6 (-18.4, -10.8) |
| Imaging use | -3.8 (-8.2, 0.7) | -12.5 (-18.4, -6.6) | -0.5 (-3, 2.1) | -6.7 (-10.6, -2.8) |
| MRI/CT for back pain | -0.7 (-5.3, 3.9) | 1.2 (-3.7, 6) | -1.9 (-5.3, 1.5) | -1.1 (-4, 1.7) |
| Radiograph for back pain | 0.8 (-5.9, 7.5) | -1.9 (-9.6, 5.8) | -2.5 (-4.1, -1) | 3.9 (-0.7, 8.5) |
| MRI/CT for headache | -2.7 (-3.6, -1.8) | 12.5 (6, 18.9) | -2.7 (-5.2, -0.1) | -0.4 (-1.9, 1.1) |

Abbreviation: NSAID, nonsteroidal anti-inflammatory drug; MRI, magnetic resonance imaging; CT, computed tomography.

To quantify differences in the utilization of both general health care services and low-value services across Asian subpopulation groups, we ran a linear probability model after controlling for individual-level characteristics and year-fixed effects. Using the marginal effects from these models, we estimated the mean adjusted values of the outcomes for each Asian subpopulation group while holding all other variables constant except the variable of interest. Furthermore, we estimated the adjusted differences in outcomes among Asian subpopulation groups relative to the White population.

Appendix Table C. Adjusted differences in use of low-value care among Latino subpopulation groups relative to White population (after adjusting for all factors).

|  | Adjusted differences (relative to white adults), percentage points (95% CI) | | | | | |
| --- | --- | --- | --- | --- | --- | --- |
| Outcome | Central/South American | Cuban | Dominican | Mexican | Other Hispanic | Puerto Rican |
| Cancer screening | 14.5 (11, 18) | 17.2 (10.1, 24.4) | 9.6 (9.1, 10.2) | 0.8 (-0.6, 2.2) | -2.9 (-9.4, 3.7) | 10.6 (3, 18.2) |
| Cervical cancer screening | 17.3 (13, 21.7) | 24.5 (16.9, 32.1) | 10.1 (6.4, 13.8) | 5.7 (3, 8.4) | 1.9 (-4.7, 8.6) | 13.8 (10.3, 17.3) |
| Colorectal cancer screening | -1.9 (-2.9, -0.9) | 2.7 (-6.1, 11.4) | 0.4 (-0.8, 1.7) | 0.5 (-0.4, 1.4) | -2.2 (-3, -1.4) | 3.5 (-1.4, 8.4) |
| Prostate cancer screening | 5.9 (-5.9, 17.6) | 5 (-27.7, 37.6) | 3.4 (-20.2, 27) | -8.2 (-9.8, -6.6) | -9.5 (-32.4, 13.4) | 1.4 (-26.2, 28.9) |
| Medication use | -5.3 (-8.6, -2) | -9.3 (-13.7, -5) | -6.3 (-10.6, -2.1) | -6.8 (-7.8, -5.8) | -4.2 (-8.9, 0.6) | -4.2 (-5.8, -2.6) |
| Antibiotics for acute upper respiratory infection | -7.2 (-13, -1.5) | -7.7 (-14.4, -1.1) | -7.7 (-19.4, 4) | -0.2 (-1.9, 1.5) | 4.3 (-3.2, 11.8) | -4.4 (-10.6, 1.9) |
| Antibiotics for influenza | 8.4 (6.3, 10.5) | 4.5 (-5.6, 14.7) | 5.6 (-2.8, 14) | 3.4 (2.3, 4.4) | 3 (-1.9, 7.9) | -0.2 (-2.6, 2.1) |
| Benzodiazepine for depression | -2.7 (-12.2, 6.9) | 13.8 (1.2, 26.4) | -9.1 (-21, 2.9) | -4.2 (-5.2, -3.2) | -4.7 (-10.8, 1.5) | -2.4 (-8.3, 3.4) |
| Opioid for back pain | -4.2 (-6.3, -2.1) | -14 (-15.9, -12.2) | -7.9 (-12.6, -3.2) | -6 (-9.6, -2.5) | -1.6 (-9.7, 6.5) | -2.2 (-3.6, -0.7) |
| Opioid for headache | -1.1 (-2.8, 0.6) | 2.7 (1.8, 3.5) | 2.7 (-1.1, 6.4) | -0.8 (-2.2, 0.7) | -1.6 (-3.2, 0) | -2.1 (-2.9, -1.2) |
| NSAID use for hypertension, heart failure, or kidney disease | -5.3 (-8.6, -2) | -9.3 (-13.7, -5) | -6.3 (-10.6, -2.1) | -6.8 (-7.8, -5.8) | -4.2 (-8.9, 0.6) | -4.2 (-5.8, -2.6) |
| Imaging use | -5.5 (-8.4, -2.5) | -2.2 (-5.9, 1.5) | 2.6 (-1.7, 6.9) | -3.3 (-5.9, -0.8) | 1.5 (-2.9, 5.9) | -1.4 (-3.4, 0.6) |
| MRI/CT for back pain | -1.6 (-2.4, -0.8) | 0.1 (-4.2, 4.3) | -4.9 (-7.1, -2.6) | -3 (-4.1, -1.9) | -2.7 (-4.4, -1) | -2.4 (-3.5, -1.4) |
| Radiograph for back pain | 3.4 (1.9, 5) | 0 (-0.5, 0.4) | 1.7 (-2, 5.3) | -0.1 (-2.6, 2.3) | -1.1 (-4.4, 2.1) | -0.1 (-3.8, 3.7) |
| MRI/CT for headache | 4.3 (-1.5, 10.2) | 1.2 (-3.1, 5.4) | -1.8 (-4, 0.5) | 0.3 (-1.3, 1.9) | -2.3 (-5.2, 0.6) | 1.8 (-4.9, 8.5) |

Abbreviation: NSAID, nonsteroidal anti-inflammatory drug; MRI, magnetic resonance imaging; CT, computed tomography.

To quantify differences in the utilization of both general health care services and low-value services across Latino subpopulation groups, we ran a linear probability model after controlling for individual-level characteristics and year-fixed effects. Using the marginal effects from these models, we estimated the mean adjusted values of the outcomes for each Latino subpopulation group while holding all other variables constant except the variable of interest. Furthermore, we estimated the adjusted differences in outcomes among Latino subpopulation groups relative to the White population.

Appendix Table D. Adjusted differences in use of low-value care among Asian subpopulation groups relative to White population (after adjusting for age, sex, and chronic conditions).

|  | Adjusted differences (relative to White adults), percentage points (95% CI) | | | |
| --- | --- | --- | --- | --- |
| Outcome | Asian Indian | Chinese | Filipino | Other Asian |
| Cancer screening | -5.2 (-15.9, 5.4) | -3.7 (-12.6, 5.2) | -6.8 (-12.1, -1.5) | -6.2 (-19, 6.7) |
| Cervical cancer screening | 2.8 (-8.3, 14) | -4.2 (-7, -1.4) | -5.4 (-8.6, -2.2) | -2.2 (-8.4, 4) |
| Colorectal cancer screening | 0.3 (-3.4, 4.1) | -3.5 (-4.9, -2.1) | 0.3 (-5.6, 6.2) | 2 (-2.7, 6.6) |
| Prostate cancer screening | -15.1 (-31.3, 1.1) | -0.7 (-20.3, 18.8) | -6.7 (-27.1, 13.7) | -17.1 (-45.5, 11.3) |
| Medication use | -11.5 (-14.7, -8.3) | -5.1 (-7.1, -3.1) | -9.3 (-13, -5.6) | -14.6 (-18.4, -10.8) |
| Antibiotics for acute upper respiratory infection | -11.1 (-17, -5.3) | -12.9 (-23.2, -2.6) | -9.5 (-13.8, -5.1) | -11.9 (-15.8, -7.9) |
| Antibiotics for influenza | 5 (-0.8, 10.8) | -1 (-4.2, 2.2) | 7.8 (-2.6, 18.3) | 0.9 (-5.6, 7.3) |
| Benzodiazepine for depression | -10.5 (-19.5, -1.6) | -12.8 (-16.1, -9.5) | -15.6 (-25, -6.1) | -12.4 (-16, -8.7) |
| Opioid for back pain | -7 (-11.4, -2.7) | -10.7 (-13.9, -7.6) | -9.1 (-16.1, -2.2) | -7 (-9.7, -4.2) |
| Opioid for headache | -2 (-2.7, -1.3) | -3.1 (-4.6, -1.7) | -2.4 (-3.1, -1.6) | 6.4 (4.3, 8.4) |
| NSAID use for hypertension, heart failure, or kidney disease | -11.5 (-14.7, -8.3) | -5.1 (-7.1, -3.1) | -9.3 (-13, -5.6) | -14.6 (-18.4, -10.8) |
| Imaging use | -3.8 (-8.2, 0.7) | -12.5 (-18.4, -6.6) | -0.5 (-3, 2.1) | -6.7 (-10.6, -2.8) |
| MRI/CT for back pain | -0.7 (-5.2, 3.7) | 0.5 (-4.2, 5.2) | -2.2 (-5.3, 1) | -1.5 (-3.9, 0.9) |
| Radiograph for back pain | 1.2 (-5.6, 8.1) | -2.4 (-9.5, 4.8) | -2.6 (-4.3, -0.9) | 3.1 (-2.1, 8.3) |
| MRI/CT for headache | -2.3 (-3, -1.6) | 12.5 (5.2, 19.9) | -2.7 (-4.5, -0.9) | -0.9 (-2.3, 0.5) |

Abbreviation: NSAID, nonsteroidal anti-inflammatory drug; MRI, magnetic resonance imaging; CT, computed tomography.

To quantify differences in the utilization of both general health care services and low-value services across Asian subpopulation groups, we ran a linear probability model after controlling for individual-level characteristics and year-fixed effects. Using the marginal effects from these models, we estimated the mean adjusted values of the outcomes for each Asian subpopulation group while holding all other variables constant except the variable of interest. Furthermore, we estimated the adjusted differences in outcomes among Asian subpopulation groups relative to the White population.

Appendix Table E. Adjusted differences in use of low-value care among Latino subpopulation groups relative to White population (after adjusting for age, sex, and chronic conditions).

|  | Adjusted differences (relative to white adults), percentage points (95% CI) | | | | | |
| --- | --- | --- | --- | --- | --- | --- |
| Outcome | Central/South American | Cuban | Dominican | Mexican | Other Hispanic | Puerto Rican |
| Cancer screening | 10.3 (5.7, 14.9) | 15.2 (6.5, 23.9) | 4.1 (2.9, 5.3) | -6.9 (-9.7, -4.2) | -6.4 (-12.8, 0) | 6.7 (-1.5, 15) |
| Cervical cancer screening | 13 (9.5, 16.6) | 23 (17, 28.9) | 5.4 (2, 8.7) | -0.2 (-5.5, 5.1) | -0.9 (-8.2, 6.4) | 10.7 (5.1, 16.3) |
| Colorectal cancer screening | -1.4 (-2, -0.9) | 3.6 (-4.2, 11.5) | 1.6 (0.8, 2.4) | -0.4 (-1.6, 0.8) | -2.8 (-3.2, -2.5) | 3.9 (-0.8, 8.6) |
| Prostate cancer screening | 4.8 (-2.6, 12.1) | 2.2 (-22.2, 26.5) | -2.5 (-5.7, 0.7) | -17.8 (-28.4, -7.2) | -14.2 (-39.3, 10.9) | -5.5 (-24.8, 13.8) |
| Medication use | -13 (-16.1, -10) | -17 (-19.9, -14.1) | -15.8 (-22.1, -9.6) | -16 (-17.1, -14.9) | -7.8 (-12.1, -3.4) | -10.2 (-12.2, -8.1) |
| Antibiotics for acute upper respiratory infection | -9.6 (-14.1, -5.1) | -5.1 (-11.5, 1.3) | -8.5 (-19.1, 2.1) | -5.8 (-7.4, -4.3) | 0.7 (-9.2, 10.6) | -5.5 (-11.4, 0.4) |
| Antibiotics for influenza | 8 (6.8, 9.3) | 5.4 (-2.9, 13.6) | 4.1 (-3.6, 11.8) | 1.9 (1.4, 2.3) | 2.5 (-1.6, 6.6) | -0.7 (-2.7, 1.3) |
| Benzodiazepine for depression | -2.7 (-12.1, 6.8) | 17.1 (4.9, 29.2) | -7.4 (-18.5, 3.7) | -4.8 (-5.6, -4) | -4.4 (-11.2, 2.3) | 0.2 (-7.1, 7.5) |
| Opioid for back pain | -2.4 (-4, -0.8) | -8.4 (-8.8, -7.9) | -2.9 (-7.2, 1.3) | -2.1 (-5, 0.7) | 0.7 (-8.1, 9.5) | 2.3 (0.2, 4.4) |
| Opioid for headache | -0.6 (-2.5, 1.2) | 3.1 (1.3, 4.9) | 3.4 (-0.2, 7.1) | 0.3 (-0.7, 1.3) | -1.3 (-2.8, 0.2) | -1.5 (-2.8, -0.3) |
| NSAID use for hypertension, heart failure, or kidney disease | -13 (-16.1, -10) | -17 (-19.9, -14.1) | -15.8 (-22.1, -9.6) | -16 (-17.1, -14.9) | -7.8 (-12.1, -3.4) | -10.2 (-12.2, -8.1) |
| Imaging use | -8.6 (-10.9, -6.3) | -4.3 (-8.4, -0.2) | -1.8 (-5, 1.5) | -7.2 (-9.9, -4.5) | 0.4 (-4.7, 5.4) | -3.5 (-5.5, -1.6) |
| MRI/CT for back pain | -1.3 (-2.3, -0.3) | 1.4 (-1.1, 4) | -4.4 (-5.9, -2.9) | -3 (-4.3, -1.6) | -2.4 (-3.9, -0.9) | -2 (-2.7, -1.3) |
| Radiograph for back pain | 3.5 (2, 5.1) | 0.8 (0.6, 1) | 1.4 (-1.4, 4.3) | -0.5 (-2.8, 1.7) | -1.4 (-5, 2.3) | -0.4 (-2.7, 1.8) |
| MRI/CT for headache | 4.2 (-0.9, 9.4) | 0.3 (-3.4, 4) | -1.9 (-4.2, 0.4) | -0.2 (-1.8, 1.4) | -2.7 (-4.3, -1) | 1.9 (-4.9, 8.8) |

Abbreviation: NSAID, nonsteroidal anti-inflammatory drug; MRI, magnetic resonance imaging; CT, computed tomography.

To quantify differences in the utilization of both general health care services and low-value services across Latino subpopulation groups, we ran a linear probability model after controlling for individual-level characteristics and year-fixed effects. Using the marginal effects from these models, we estimated the mean adjusted values of the outcomes for each Latino subpopulation group while holding all other variables constant except the variable of interest. Furthermore, we estimated the adjusted differences in outcomes among Latino subpopulation groups relative to the White population.
